# On the Recent Outbreak of “Febris Nigra” in Dublin

**Published:** 1868-05

**Authors:** Robert D. Lyons

**Affiliations:** Dublin


### On the recent Outbreak of "Febris Nigra" in Dublin.

BY ROBERT D. LYONS, M. B., DUBLIN.

(British Medical Journal, July 6th, 1867.)

At a meeting of the Epidemiological Society on July 1st, Dr. Lyons communicated the notes of several cases, and exhibited specimens of the spinal cord from two cases. He proposed to designate the disease "*febris nigra.*" He believed that the malady is allied to yellow fever, cholera, and in a certain sense to typhus, and to other diseases in which, without necessary physical lesion, a profound prostrating effect is exercised on the nervous centres, and through these on the blood. As to the causation of the disease, he referred to the general sanitary state of Dublin, which he described as bad as bad can be. When cases of this disease occurred in 1866, Dr. Lyons called the attention of the Lord-Lieutenant and local authorities to them as an indication of a marked change in the epidemic constitution, and predicted an epidemic possibly of cholera, which came in due time. This year he made a similar prediction, and he remarked that at least one case of cholera had already occurred in Dublin.

Dr. Edwards Crisp related the circumstances attending a case of purpuric fever which had occurred at Chelsea on the 16th June. There was no doubt it was a case identical in nature with the cases described by Dr. Mapother, Dr. Marston and Dr. Lyons. He dwelt upon the purpuric affection observed among pigs in Dublin, and offered some suggestions for the experimental study of both the human and brute purpuric disorder.

Mr. Heather Bigg briefly stated certain facts respecting a rapidly fatal case of purpuric disorder which had occurred on the 26th ult. in Bethnal Green, the circumstances having been mentioned to him in a recent conversation with Mr. Humphrey, the coroner.

Dr. J. Burdon Sanderson expressed some surprise at the suggestion of Dr. Mapother, that there might be two diseases confounded together in the recent outbreak in Dublin—a malignant typhus and cerebro-spinal arachnitis. Both forms of disorder insensibly passed the one into the other; the analogy of the virulent form with typhus had no existence in fact either as to the course of the disease, eruption, or post-mortem appearance. The couclusion rather was that we had to do with different grades of the same disease. He expressed regret at the paucity of post-mortem examinations, but thought that the general symptoms afforded strong grounds for the belief that the disease was identical with cerebro-spinal meningitis which had been observed in Prussia in 1865. He read a note from Dr. Stokes, descriptive of the different forms of the disease which had occurred in Dublin.

Dr. Marston remarked that the opinion that the malady might be typhus in a scurvy-affected subject clashed altogether with experience. Typhus in the scurvy-stricken had been largely observed in the Crimean war, but no such symptoms had been manifested as were observed in the purpuric fever of Dublin. There was an entire absence of evidence to attach any of the phenomena of the disease to scurvy, and the opportunity for ascertaining the fact had been largely furnished during the late war in the United States. The question was one, however, not to be lost sight of.

Dr. Camps pointed out the dissimilarity of the disease from the specific fevers with which we are acquainted.

Dr. A. P. Stewart, as showing the difference of typhus from the malady in Dublin, remarked that the latter carried off large numbers of well-to-do people. More than one-third of the cases in Dublin, he observed, occurred in the better parts of the city. In America, also, the well-to-do suffered equally perhaps with the ill-to-do. No proper typhus had, moreover, been noted. In scurvy no form of disease like the Dublin disease had hitherto been noticed; and in diseases affecting persons affected with scurvy no like series of symptoms had been recorded. Our familiarity with the course of the latter disease, under many and varied circumstances, would lead to the conclusion that the purpuric spots in the Dublin disease owned some other origin. Dr. Stewart had seen within the last two years, in many cases of disease, and especially in rheumatism, copious dark purpuric blotches. He had noticed the same phenomena in 1858. During the last three or four years, in fact, he had observed a purpuric tendency in several maladies; and he was inclined to think that in the Dublin affection, the purpuric spots were not characteristic of the disease, while the lesion of the cerebro-spinal centres was. In the majority of the cases he had seen, there were indications of morbid action in those centres.

Dr. Yandell (United States) observed that, although he had not had an opportunity of witnessing cerebro-spinal meningitis, the spotted fever of some American authors, he had during the war seen thousands of cases of scurvy among the troops at Corinth. Nothing, however, like "spotted fever," or comparable to that disease, came under his notice, although the disease so called prevailed in other localities, and especially among the Federal forces. Very many men were lost from scorbutic diarrhœa. He gave some details, also, of epizootics in Tennessee.

Dr. Bowen referred to the title "Black Death," which had been adopted by some to designate the malady in Dublin, and observed that it was a singular coincidence that the same plague was reported to have broken out among some of the tribes on the banks of the Euphrates.

Dr. Trader (United States) had seen several sporadic cases of cerebro-spinal meningitis, but had never had the opportunity of witnessing the disease in an epidemic form. He had been led to form the opinion that the petechial spots were not characteristic of, although frequently accompanying, the disease.

Dr. Jenner remarked, as regards typhus and the Dublin disease, that, while the latter affected children much and was very fatal to them, children rarely suffered from typhus, and the latter disease still more rarely killed them. The difference was startling.—Again, in typhus, a deposit in lymph about the brain was so rare an occurrence that he had never seen a case in which it had occurred; such a deposit appeared to be characteristic of the fully-developed Dublin disease. The latter disease, in fact, taking it to be a disease *sui generis*, had a close analogy to all diseases of its order. It will kill suddenly; so will small-pox and scarlet fever; typhus rarely. And when the last named diseases kill suddenly, as in the Dublin disease, they exhibit much petechial eruption, purpuric spots and blotches, and even hemorrhage from the nose, mouth and bowels. If the blood-poison kills the patient at once, the special lesion of the disease is masked or undeveloped. If the blood-poison do not kill at the outset, the characteristic lesion is developed, and should the disease in the end prove fatal, kills the patient. The names which had been given to the Dublin disease since its appearance were gratifying, as far as they indicated the activity of thought with which the new to us and as yet ill-comprehended malady was regarded. The name by which the disease was best known (cerebro-spinal meningitis) was bad, but the new names proposed were worse. "Purple fever," "purpuric fever," were names better applicable to certain forms of continued fever, and which it would be impossible to limit to the disease under consideration. The terms "malignant purpuric fever" and "neuro-purpuric fever" exaggerated the erroneousness of the previous terms. "Cerebro-spinal typhus" was a still worse form, as it was undesirable to attach a definitely understood word like "typhus" to a disease which was imperfectly known, and which had certainly little in common with typhus. "Blaek fever" was an equally unsatisfactory name, and might with as much justiee be applied to other forms of disease.—*Half-Yearly Abstract.*

